# Correlations between Clinic Preferences and Alcohol Use Disorder: an Alcohol Cohort Study in Northern Taiwan in 2022

**DOI:** 10.1192/j.eurpsy.2024.846

**Published:** 2024-08-27

**Authors:** W.-Y. Su, S.-C. Wang, S.-Y. Yeh

**Affiliations:** ^1^National Yang Ming Chiao Tung University, Taipei City; ^2^Department of Psychiatry, Taoyuan General Hospital, Taoyuan City, Taiwan, Province of China

## Abstract

**Introduction:**

Chronic alcoholism can result in severe liver conditions such as fatty liver disease and cirrhosis, potentially leading to life-threatening complications and premature death.

**Objectives:**

This study investigated the age-sex distribution of patients with alcohol addiction and aimed to identify differences in clinic department preferences based on their principal and additional diagnoses in Taiwan, in 2022.

**Methods:**

We conducted a comprehensive analysis of the diagnostic patterns of 334 patients with alcohol addiction from the Taoyuan General Hospital, Ministry of Health and Welfare.

**Results:**

**Figure 1** depicts patient demographics, highlighting 297 male and 37 female patients with alcohol-related disorders. Males aged 41-60 years were particularly dominant, as shown in **Figure 2**. Principal diagnoses, including alcoholic liver disease and acute pancreatitis, are detailed in **Table 1**. Additional diagnoses, such as chronic pancreatitis and esophageal varices, are presented in **Table 2**. For departmental preferences, **Table 3** reveals the Gastrointestinal (GI) department as the top choice, followed by Kidney, Neurological, and Cardiovascular/Chest.

**Table 1.** Top 5 Principal Diagnoses of Alcohol Addiction Patients.

ICD-10-CM	Principle diagnosis	Times	Rank
K70	Alcoholic liver disease	43	1
K85	Acute pancreatitis	27	2
F10	Alcohol related disorders	18	3
A41	Other sepsis	14	4
K86	Other chronic pancreatits	11	5

**Table 2.** Top 5 Additional Diagnoses of Alcohol Addiction Patients.

ICD-10-CM	Additional diagnosis	Times	Rank
F10	Alcohol related disorders	40	1
K86	Other chronic pancreatits	18	2
I85	Esophageal varices	16	3
K70	Other sepsis	16	
E87	Other disorders of fluid, electrolyte and acid-base balance	15	4
R65	Symptoms and signs specifically associated with systemic inflammation and infection	10	5

**Table 3.** Top 5 Departments for Alcoholism Patient Presentation.

Department	Times	Rank
Gastrointestinal	162	1
Kidney	39	2
Neurological	25	3
Cardiovascular Chest	15	4

**Image:**

**Figure:**
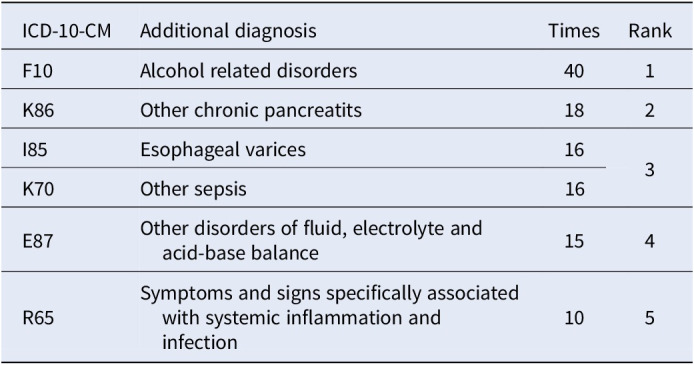

**Image 2:**

**Figure:**
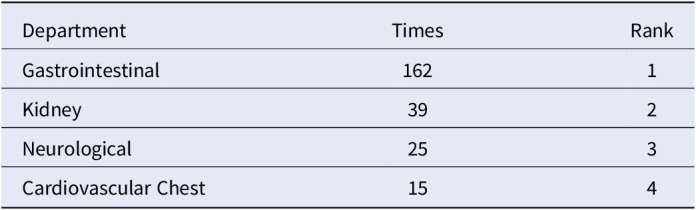

**Conclusions:**

The study revealed that patients with alcohol addiction often delay seeking psychiatric help instead of presenting for medical care only after liver or gastrointestinal complications occur. This underscores the crucial need for better health education regarding the relationship between alcohol addiction and liver disease. Prompt recognition and early intervention for substance addiction can significantly reduce these risks and improve patient outcomes.

**Disclosure of Interest:**

None Declared

